# Dietary supplement use is common in older adult drivers: an analysis from the AAA LongROAD study

**DOI:** 10.1186/s12906-024-04623-x

**Published:** 2024-08-30

**Authors:** Ryan Moran, Sara Baird, Carolyn G. DiGuiseppi, David W. Eby, Sarah Hacker, Chelsea Isom, Vanya Jones, Kelly C. Lee, Guohua Li, Lisa J. Molnar, Rudy Patrick, David Strogatz, Linda Hill

**Affiliations:** 1 Herbert Wertheim School of Public Health and Longevity Science, University of California San Diego, 9500 Gilman Drive, La Jolla, CA 92093 USA; 2 Colorado School of Public Health, University of Colorado Anschutz Medical Campus, 13001 E 17th Place, Campus Box B119, Aurora, CO 80045 USA; 3 College of Engineering, University of Michigan Transportation Research Institute, 2901 Baxter Rd, Ann Arbor, MI USA; 4 Department of Health, Behavior and Society, Bloomberg School of Public Health, Johns Hopkins University, 624 N. Broadway, Baltimore, MD 21205 USA; 5 Skaggs School of Pharmacy and Pharmaceutical Sciences, University of California San Diego, 9500 Gilman Drive, La Jolla, CA MC 0657 USA; 6 Department of Epidemiology, Mailman School of Public Health, Columbia University, 622 West 168th Street, New York, NY PH5-534 USA; 7 Department of Anesthesiology, Vagelos College of Physicians and Surgeons, Columbia University Irving Medical Center, 622 West 168th Street, New York, NY PH5-534 USA; 8 University of Michigan Transportation Research Institute, 2901 Baxter Road, Ann Arbor, MI 48109-2150 USA; 9 Bassett Research Institute, Bassett Healthcare Network, One Atwell Road, Cooperstown, NY 13326 USA

**Keywords:** Older adult, Dietary supplement, Supplement, Polypharmacy

## Abstract

**Background:**

Dietary supplement (DS) use is common and increasing among older adults, though much data available on use frequencies are from surveys and performed cross-sectionally. This paper sought to assess the frequency and pattern of dietary supplement use among older adults over time.

**Methods:**

A secondary analysis of data from the AAA LongROAD study, a longitudinal prospective cohort study of older adult drivers, using data from baseline and the first two years of follow up included a total of 2990 drivers aged 65–79 years recruited at five study sites across the US from July 2015 to March 2017. Participants underwent baseline and annual evaluations, which included a “brown bag” medication review. DS were identified and categorized according to type and key components. Prevalence and pattern of DS use over time and relationship to demographics were measured with frequency and Chi squared analyses.

**Results:**

84% of participants took at least one dietary supplement during the 2-year study period, and 55% of participants continually reported use. DS accounted for approximately 30% of the total pharmacologic-pill burden in all years. Participants who were White non-Hispanic, female, 75–79 years of age at baseline, and on more non-supplement medications took significantly more dietary supplements (*P* < 0.05). Vitamin D, multivitamins, calcium, and omega-3 formulations were the most common supplements, with stable use over time. Use of individual herbal supplements and cannabis products was uncommon (< 1% participants per year).

**Conclusions:**

DS use among older adults is common and relatively stable over time and contributes to polypharmacy. In clinical settings, providers should consider the influence of DS formulations on polypharmacy, and the associated cost, risk of medication interactions, and effect on medication compliance.

**Supplementary Information:**

The online version contains supplementary material available at 10.1186/s12906-024-04623-x.

## Background

In the last decade, the percentage of Americans aged 65 or older has increased by over 40%, [1, 2] with older adults representing approximately 17.1% of the population in 2021 [1]. Over 30% of older adults experience polypharmacy, defined as five or more co-prescribed medications [3], which is associated with poorer health outcomes and increased frailty in this population [4–6]. While these correlations are likely bidirectional, polypharmacy in older adult populations is a source of ongoing concern and study.

Dietary supplement (DS) formulations are products intended to supplement diets, and include vitamins, herbal and botanical substances or compounds, amino acids, minerals, and probiotics. While DS forms are heterogeneous, the primary reported reason or motivator for use is to improve, supplement, or maintain health [7]. Despite overall lack of evidence of effectiveness [8], supplement use has increased in the last several decades in the United States (U.S.), and the rate of DS use increases with age [9]. Survey data from the National Health and Nutrition Examination Survey suggest that around 74% of U.S. adults over the age of 60 use some form of DS [10], while other surveys suggest even higher rates of use [3, 11]. These and other studies have identified multivitamin, vitamin D, and omega-3 fatty acids as the most commonly reported supplements [3, 10, 12].

The use of DS formulations has clinical implications that are underappreciated and often unaccounted for in healthcare. While the decision between a healthcare provider and a patient to start a medication is often balanced against benefit and risk, healthcare providers are infrequently trained on the risks of DS forms, including medication interactions and adverse effects. Despite this, providers often suggest DS formulations to patients [13]. In addition, many patients start a DS on their own, and do not report this to medical providers [14].

It remains poorly understood which and how frequently supplements are used in real-world settings, given most existing data are done cross-sectionally and based on surveys. Further, there remains little data regarding their use over time. A major concern from these limitations is that the interpretation of data from existing studies is variably limited by use of survey/self-report, cross-sectional design, and interpretation of formulations with multiple constituent compounds to fully characterize exposures of DS that may or may not have been considered, such as in a name brand that carries 3 different ingredients. Given ongoing concerns with polypharmacy in older adults, the frequencies of these agents and their relative contribution to the total pharmacologic daily pill burden remain a subject of interest.

The American Automobile Association (AAA) Longitudinal Research on Aging Drivers (LongROAD) study is a multi-center prospective cohort study of older drivers in the U.S., which aims to better understand how medical, behavioral, social, technological, and environmental factors affect driving safety among adults over the age of 65. The LongROAD study enrollment included brown-bag medication review at baseline and annually, where participants were instructed to bring in/record all regularly taken compounds, both those prescribed and those obtained over-the-county. This systematic characterization of medications, and meticulous coding of all compounds taken regularly by participants provides an opportunity to understand the naturalistic exposures of both medications and non-prescribed formulations taken throughout this study period. Previous analysis evaluated medication use and outcomes by medication class, noting high numbers of unclassifiable medications without further exploration of that subset [15]. The aims of this analysis are to (1) describe the frequencies of medication and supplement use in older adult drivers with a focus on the relative burden of dietary supplements on polypharmacy; (2) to describe the most common formulations, and the most common constituent DS components used in this population, (3) describe the trends of dietary supplement use over time, and (4) explore inferential statistics to evaluate associations between demographics and DS use propensity.

## Methods

The LongROAD study methods have been described in detail elsewhere [16]. Briefly, the LongROAD study was a multi-center longitudinal prospective cohort that enrolled older adult drivers aged 65–79 years from five study locations (Ann Arbor, MI; Baltimore, MD; Cooperstown, NY; Denver, CO; and San Diego, CA) between July 2015 and March 2017, funded by the AAA Foundation. Drivers could enroll if they were the primary driver of their non-commercial vehicle (e.g. car, truck, or sport utility vehicle). Institutional Review Board approval was obtained at each study site, and written informed consent was obtained for all participants. At baseline, demographics were collected by study-site.

During enrollment and at year two, participants completed in-person “brown-bag medication reviews,” where participants were asked to bring in all prescription and non-prescription medications and dietary supplements, which were then documented by research staff. In year one, the medication review was conducted by phone.

All medications were recorded into an online database by on-site research assistants, and later coded according to the American Society of Health-System Pharmacists (AHFS) Pharmacologic-Therapeutic Classification System [17]. The AHFS classification system allows grouping of medications with similar pharmacologic, therapeutic and/or chemical characteristics using a four-tier hierarchy, where each subsequent tier provides more detailed information about the medication group.

Participant medications were initially classified by a clinical psychiatric pharmacist and confirmed by physician review. When spelling errors occurred, the most likely medication was coded, and when medication contents or type could not be determined (due to ambiguous naming or misspellings), the item was left as non-classified. Non-classified medications which do not fall into the AHFS classification system also included food-like items (e.g., turmeric, mushroom extract), homeopathic products, and other supplements (e.g., saw palmetto, lutein).

Medications were considered for inclusion as a DS by being in the following medication classes: (1) Non-classified medication, (2) AHFS Classification Tier 88 (Vitamins), or (3) Tier 40:12 (Electrolyte, Caloric, and Water Balance - Replacement Preparations). For tiers that contained prescription medications (Tier 40:12), only those that were not prescription or were clearly over the counter without clear pharmacologic purpose after review from 2 clinicians were included. Once the DS database was established, a team of two experienced clinicians independently reviewed entries to confirm medication identification as well as assign grouping for purposes of this analysis. A list and description of AHFS Classifications for example DS and their subsequent supplement categorization groups can be found in Table 1.

**Table 1:** **List and description of AHFS classifications and supplement categorizations**

AHFS Classification Tier I:	AHFS Classification Tier II:	Example Medication Entry	Categorization
40 (Electrolyte, Caloric, and Water Balance)	40:12 (Replacement preparations)	Citracal Calcium with D3	Calcium Vitamin D
88 (Vitamins)	88:04 (Vitamin A)	Vitamin A	Vitamin A
	88:08 (Vitamin B Complex)	Cyanocobalamin Vitamin B12	Vitamin B12
	88:12 (Vitamin C)	Chewable Vitamin C	Vitamin C
	88:16 (Vitamin D)	Dry Vitamin D3 Cholecalciferol Supplement	Vitamin D
	88:20 (Vitamin E)	Swanson Mixed Tocopherols	Vitamin E
	88:24 (Vitamin K)	Menaquinone 7 Vitamin K2	Vitamin K
	88:28 (Multivitamin Preparations	Centrum Silver	Multivitamin
Unclassified	N/A	Alpha Lipoic CoQ10	Lipoic acid CoEnzyme Q
		Ocuvite Oral Eye Supplement	Eye vitamin
		Nature Made Super B Complex	B combo
		Salmon Oil	Omega 3
		Carboxymethylcellulose sodium	Artificial tears
		Saline Nasal Spray	Topical
		Beano	Digestive enzymes
		Probiotic acidophilus	Probiotic
		Chromium	Elemental mineral
		CBD oil	Cannabis product
		Free form L Lysine	Amino acid
		Green Tea Extract	Other
		Dietary Supplement	Undetermined

Groupings included:

- Individual supplements by name (e.g., Vitamin A, Turmeric, Omega 3).
- Specific supplement category (includes ‘Cannabis product’, ‘Digestive enzymes’, ‘Probiotic’, ‘Elemental mineral’, ‘Amino acid’, ‘Artificial tears’).
- B vitamin combination (formulations with at least 2 B-vitamins without other components).
- ‘Multivitamin’ (formulations identified as “multivitamin” by name, or formulation with at least five component vitamins and minerals).
- ‘Eye vitamin’ (oral vitamin combination products identified for ophthalmologic benefit; includes lutein and zeaxanthin products).
- ‘Topical’ (unmedicated topical preparations e.g., saline nasal spray).
- Other (includes homeopathic preparations and less common herbal compounds e.g., horse chestnut, green tea extract, etc.)
- Undetermined (components unknown, such as “Nerve Support Formula”, “Sam’s Club” etc., but likely to be a dietary supplement).

Given no widely available accepted definition, multivitamins were identified by either being entered clearly as one, or by combining compounds that had 5 or more different vitamins or minerals, given the incredible heterogeneity in the dataset allowing for categorization [8]. Supplements that had four or fewer clearly identifiable ingredients, including name-brand products, were separated by constituent supplements/vitamins (for example, participants taking “Calcium/Vitamin D” combination supplement was coded as “Vitamin D” and “Calcium”; “Move Free Advanced” was coded as ‘Chondroitin’, ‘Glucosamine’, ‘MSM’, and Vitamin D’). These separated compounds were also evaluated to better understand how individual vitamin compound frequencies changed throughout this cohort and to understand the degree of duplicative use of some DS formulations. Items that were AHFS-unclassified but not dietary supplements (e.g. oxygen) were excluded from analysis. Non-medicated topical agents were purposefully included if there was a theoretical use to improve health, though not traditionally considered dietary supplements (e.g. topical nasal saline, arnica gel). The included topical agents compromised less than 1% of the total DS data.

### Analysis

Demographic and medication data from baseline, year 1, and year 2 were included for analysis. At each time point, participants were categorized based on DS use as either users (i.e., reported ≥ 1 supplement) or non-users (i.e., reported 0 supplements). Additionally, the total number of medications and DS forms were calculated. Frequencies were obtained for both aggregate formulations (each item entered during brown bag review) as well as by their individual constituent components, as noted above. These individual components were reported at the unique participant level (number of unique participants using a given DS or DS component at all time-points). Polypharmacy was defined as at least five co-prescribed medications, excluding DS [18]. Pill burden was defined as the total number of medications a specific participant was on regardless of formulation (e.g. prescribed or DS).

To explore DS use consistency, a user’s propensity to utilize any DS across time, we calculated uses of individual components regardless of changes in DS formulation. Consistent users were defined as those taking a specific DS at all time points (baseline, year 1, year 2), and intermittent users were defined as use during 1–2 time points. Consistent non-users were defined as those participants who had no DS compound at any point through our analyzed study period. Percentages calculated utilized the final enrollment number as the denominator to remain consistent despite study loss to follow up across the timeframe, which allows for comparing trends in this study period. Although study-drop out may have impacted these percentages (e.g. if those with or without DS were more or less likely to remain in the study), given the study design was related to driving behaviors, with DS collected annually but observationally, the use or non-use of these compounds were not likely to directly impact study-adherence for this exploratory analysis. Finally, we calculated DS redundancy: the percent and number of users who utilize more than one of the same DS component at the same time point in different formulations. Cross tabulations, pairwise T-tests, and chi-square analysis was utilized to evaluate associations between DS use and demographic variables, and for medication frequency use and DS utilization at the participant level, using *p* < 0.05 for statistical significance. All analyses were conducted using SAS 9.4 (SAS Institute; Cary, NC) and SPSS v. 28 (IBM, Armonk, NY).

## Results

There were 2,990 participants at baseline, 2,879 at Year 1, and 2,717 at Year 2. Across a total of 8,586 participant-measurement encounters, there were 72,339 medication entries, of which 62,814 (86.8%) were classified in AHFS, and 9,523 remained unclassified (13.2%).

A total of 21,051 medications (29.1%) were identified as a DSs, consisting of: (1) 9,238 unclassified medications; (2) 454 medications from tier 40:12 (Replacement preparations), and (3) 11,359 items from Tier 88 (Vitamins). After categorization, there were a total of 145 different supplement formulations, and 59 unique individual components (Appendix A).

### Demographics and medication use at baseline

Demographics and medication use are summarized in Table 2. Enrollment at baseline was approximately equal across each of the five study sites. Of the baseline participants, the majority were non-Hispanic White, female, and educated. Most (66.5%) were married or living with a partner, 14.9% divorced, and 12.7% widowed.

**Table 2:** Demographics of the older adult participants of the AAA LongROAD study at baseline (*N* = 2990)

	*n*	%	Total Medications and Dietary Supplements	Medications	DS
			mean (SD)	mean (SD)	mean (SD)
**Study Site**					
Denver, CO (A)	600	20.1	8.81 (5.6)^**b, c,e**^	5.85 (4.1)	2.96 (3.5)^**b, c,e**^
Cooperstown, NY (B)	601	20.1	7.66 (4.6)^**e**^	5.27 (3.5)	2.38 (2.7)^**e**^
Baltimore, MD (C)	588	19.7	7.59 (4.7)	5.39 (3.6)	2.2 (2.4)^**c, e**^
Ann Arbor, MI (D)	601	20.1	8.37 (4.8)^**e**^	5.68 (3.9)	2.68 (2.6)^**c, e**^
San Diego, CA (E)	600	20.1	7.22 (5.1)	5.45 (4.0)	1.78 (2.6)
**Age group**					
65–69 years (A)	1243	41.6	7.44 (4.8)	5.18 (3.6)	2.27 (2.4)
70–75 years (B)	1037	34.7	7.98 (5.1)^**a**^	5.62 (3.8)^**a**^	2.36 (2.8)
75–79 years (C)	710	23.8	8.70 (5.2)^**a, b**^	6.00 (3.8)^**a**^	2.70 (3.3)^**a, b**^
**Gender**					
Male (A)	1403	46.9	7.54 (4.8)	5.6 (3.8)	1.93 (2.4)
Female (B)	1587	53.1	8.28 (5.1)^**a**^	5.45 (3.9)	2.83 (3.0)^**a**^
**Race**					
White, Non-Hispanic	2557	85.6	7.97 (5.0)	5.50 (3.8)	2.47 (2.8)*
Black/African American	212	7.1	7.79 (4.9)	5.96 (4.0)	1.83 (2.5)
Hispanic	81	2.7	7.21 (4.4)	5.14 (3.4)	2.06 (2.3)
Asian	66	2.2	7.31 (5.3)	5.12 (4.0)	2.19 (2.6)
American Indian	18	0.6	9.24 (3.9)	7.35 (4.4)	1.88 (1.8)
Alaska Native, Native Hawaiian, Pacific Islander	3	0.1	5.67 (2.1)	4.67 (2.0)	1.00 (1.0)
Other	49	1.6	8.27 (5.0)	5.5 (3.4)	2.77 (2.8)
**Education**					
HS deg. or less (A)	336	11.3	7.95 (5.3)	5.74 (4.1)^**c, d**^	2.16 (2.7)
Some college (B)	528	17.7	8.34 (5.3)^**c, d**^	6.19 (4.5)^**c, d**^	2.2 (2.4)
Associate’s/Bachelor’s deg. (C)	896	30.1	7.40 (5.1)	5.07 (3.7)	2.33 (2.9)
Advanced college deg. (D)	1221	41	7.25 (5.1)	4.89 (3.7)	2.36 (2.8)
**Household income**					
Less than $20,000 (A)	134	4.7	8.56 (5.7)	6.63 (5.0)^**d, e**^	1.93 (2.2)
$20,000 - $49,999 (B)	640	22.2	8.47 (4.9)^**e**^	6.05 (3.9)^**d, e**^	2.42 (2.4)
$50,000 - $79,999 (C)	720	25	8.22 (4.9)^**e**^	5.61 (3.8)	2.61 (3.0)
$80,000 - $99,999 (D)	431	14.9	7.65 (4.5)	5.34 (3.6)	2.32 (2.5)
$100,000 or greater (E)	959	33.3	7.36 (5.0)	5.09 (3.7)	2.27 (2.7)
**Employment status^**					
No (A)	2084	69.7	7.94 (5.3)^**b**^	5.57 (4.0)^**b**^	2.37 (2.8)^**b**^
Yes (B)	904	30.3	6.66 (4.7)	4.56 (3.5)	2.10 (2.6)

a-e Superscripts reflect significance (*p* < 0.05) compared to the associated subgroup (A-E)

*: significant *p* < 0.05 against all other categories combined

^: “Did you work for pay at any time in the past month?”

The mean total pill burden was 7.55 per participant, of which approximately 30% were DS formulations (range total combined pills and DS: 0–50). Across all participants, the mean number of DS formulations was 2.29 (range: 0–47), versus a mean of 3.26 formulations among DS users. However, the distribution of DS use was heavily skewed, with 48% of DS users taking only one to two DS formulations, nearly 85% taking six or fewer DS formulations, and 97% taking fewer than 10 DS formulations suggesting few users excessively contributed to the large range for total pill burden.

Across all study sites, the average number of medications was over 5, and for those 70 years or older (Table 2); both supplement and medication use increased with age, with the oldest age group (aged 75+) using significantly (*P* < 0.05) more medications and DS forms than the younger groups. Increasing income level and education level were significantly associated with decreased mean number of medications (*p* < 0.05) but had no significant effect on DS use (*p* = 0.347 and *p* = 0.176 respectively for income and education level). There was no association between marital status and medication or supplement use (not shown in Table 2). Unemployed participants used significantly more medications and more supplements than employed participants.

### Supplement and polypharmacy Trends Over Time

A total of 2,518 (84.2%) participants reported use of at least one DS during the 2 years evaluated from the study period. Of the 2,101 participants (70.3% point prevalence) who reported DS use at the baseline medication review, 1493 (71% of initial supplement users, and 55% of all initial enrolled participants) continued to use at least one DS throughout the study period (“Consistent users”). For the 889 participants who did not report DS use at baseline, 409 did not initiate a DS throughout the study period (“consistent non-user”); there were 815 participants who reported DS use at least once but not in every point of the study (“intermittent user”) (Table 3).

Participants who had more medications were more likely to be DS users, across the length of the study (*p* < 0.001) and similarly, those identified as having polypharmacy were more likely be DS users (*p* < 0.0001) (Table 3).

**Table 3:** Medication and dietary supplement (DS) use among the older adult AAA LongROAD study participants, by year

	Baseline (*n* = 2990)		Year 1 (*n* = 2879)		Year 2 (*n* = 2717)	
	DS Nonuser *N* (%)	DS User *N* (%)	DS Nonuser *N* (%)	DS User *N* (%)	DS Nonuser *N* (%)	DS User *N* (%)
All participants	889 (29.7)	2101 (70.3)	650 (22.6)	2229 (77.4)	784 (28.9)	1933(71.1)
DS use by number of medications						
0 medications	143 (16.7)	85 (4.0)	77 (11.8)	82 (3.7)	218 (27.8)	86 (4.4)
1–4 medications	414 (33.4)	826 (39.3)	273 (42.0)	809 (36.3)	296 (37.8)	744 (38.5)
5 + medications	332 (37.3)	1190 (56.6)	300 (46.2)	1338 (60.0)	270 (34.4)	1103 (57.1)
p-value (Chi-sq)	< 0.0001		< 0.0001		< 0.0001	
	DS Consistency of Use*					
	**Non-User**		Intermittent User**		**Consistent User**	
N (%)	409 (15.1)		815 (30.0)		1493 (55.0)	

*DS consistency of use is only shown for those with complete data up to year 2

**Intermittent use is categorized as use of a DS for less than the entire 2-year period reported

### Specific supplement use

Across the 3 measurement points (Baseline, Year 1, Year 2), the three most common DS formulations were multivitamins, vitamin D, and omega-3 fatty acids. The top 15 (of 145 identified) combined DS formulations represented 79.6% of the total. Among these top 15, the majority were individual supplements (i.e., “Vitamin D”), with the exception of Calcium/Vitamin D combination (#4 of 15, 5.0% of total DS), and Chondroitin/Glucosamine (#12 of 15, 2.5% of total DS).

The top 15 individual DS forms, and the number of unique users over time, are presented in Table 4. The top 15 (of 145) individual supplement groups were selected as they represent 91.8% of the total number of DS in this cohort, and approximately 95% of supplement-using participants. After the top 15 DS, given the number of DS dropped precipitously, they are not shown in Table 4. The most common agents taken were (in order by number of users) vitamin D, multivitamin formulations, calcium, omega-3 formulations, vitamin C and glucosamine. The 15 most common supplement formulations and respective individual components (Table 4) remained relatively stable over time.

**Table 4:** Top 15 individual dietary supplements (DS) across time in the AAA LongROAD study of older adults; by total use and number of unique users

Name	Total Overall Use			Baseline		Year 1		Year 2	
	Total *N*	Percent of DS	Cumulative Percent	*N*	Unique users at baseline (%)	*N*	Unique users at year 1 (%)	*N*	Unique users at year 2 (%)
Vitamin D	4286	20.36%	20.36%	1422	1238 (41.4%)	1554	1341 (44.6%)	1310	1161 (38.8)
Multivitamin	3484	16.55%	36.91%	1172	1114 (37.3)	1269	1201 (40.2)	1043	1000 (33.4)
Calcium	2005	9.52%	46.43%	673	656 (21.9)	746	725 (24.3)	586	577 (19.3)
Omega 3	1859	8.83%	55.26%	605	581 (19.4)	700	665 (22.2)	554	534 (17.9)
Vitamin C	941	4.47%	59.73%	320	311 (10.4)	359	346 (11.6)	262	256 (8.6)
Glucosamine	929	4.41%	64.14%	317	313 (10.5)	339	334 (11.2)	273	269 (9.0)
Other	846	4.02%	68.16%	275	175 (5.8)	323	193 (6.5)	248	147 (4.9)
Vitamin B12	773	3.67%	71.83%	241	240 (8.0)	274	267 (8.9)	258	257 (8.6)
Magnesium	781	3.71%	75.54%	240	234 (7.8)	285	271 (9.1)	256	251 (8.4)
Coenzyme Q	735	3.49%	79.03%	229	229 (7.7)	262	260 (8.7)	244	243 (8.1)
Chondroitin	643	3.05%	82.08%	216	216 (7.2)	235	233 (7.8)	192	190 (6.3)
B Combo	566	2.69%	84.77%	173	172 (5.7)	216	214 (7.2)	177	174 (5.8)
Eye Vitamin	568	2.70%	87.47%	176	168 (5.6)	199	186 (6.2)	193	188 (6.3)
Probiotic	478	2.27%	89.74%	140	137 (4.6)	175	170 (5.7)	163	159 (5.3)
Potassium	429	2.04%	91.78%	147	147 (4.9)	156	151 (5.1)	126	126 (4.2)

The number of unique users versus the number of supplements (N) demonstrates a varying degree of redundancy among those who took DS formulations (e.g., where the same participant took the same DS in differing formulations, excluding individual components of multivitamins). Of the top 10 individual supplements, all showed at least some duplicative use. By far the most duplicative DS is the “Other” category, where between 57.1% and 68.7% of users who take an “Other” supplement are taking 2 or more supplements in the “Other” category. The other most common duplicative DS forms were Vitamin D (range across the study period 12.8-15.8% of participants who take Vitamin D are taking it in 2 or more forms), multivitamins (range across the study period of 4.3-5.6%), and Omega 3 (range across the study period of 3.8–5.3%).

A list of other individual DS formulations of interest given their potential clinical effect and/or perceived effects (e.g. melatonin and cannabis) is found in Table 5. Overall, the use of these supplements was very low compared with the top supplements noted above. There was little change of use over time for most supplements. Turmeric was the most common herbal supplement, and did increase in use over time, becoming the 15th most common overall individual supplement in year 2. Although use was infrequent, there was an increase in the number of users of cannabis products from 4 to 13 over the study period. The top 90 aggregated supplements, and disaggregated supplements are shown in Appendix A and B, respectively.

**Table 5:** Notable individual DS frequency of use by year of the AAA LongROAD study of older adults

	Baseline	Year 1	Year 2	Total *N* (% of total DS)
Vitamin E	110	125	98	333 (1.6%)
Turmeric/Curcumin	81	130	148	359 (1.7%)
Melatonin	79	93	85	257 (1.2%)
Cranberry	40	42	31	113 (0.5%)
Saw Palmetto	31	33	26	90 (0.4%)
Cinnamon	28	30	24	82 (0.3%)
Garlic	25	27	26	78 (0.4%)
Resveratrol	25	27	18	70 (0.3%)
Red Yeast Rice	18	19	20	57 (0.3%)
Ginkgo Biloba	15	17	12	44 (0.2%)
St John’s Wort	7	8	5	20 (0.1%)
Echinacea	7	8	4	19 (0.1%)
Cannabis	4	6	13	23 (0.1%)

## Discussion

Despite an overall lack of data supporting DS use, this study reinforced how broadly many DS formulations are used. In this population of largely educated community dwelling older adult drivers, both polypharmacy and DS use was common; approximately 84% of study participants were DS users at some point during the study period, and 55% of participants consistently listed at least one DS in their medication review from baseline to Year 2. Supplements were also a considerable portion of the total pill burden, representing approximately 30% of the total in all age groups. Participants taking more medications also used more supplements, and both medication and supplement use increased with age.

Polypharmacy is a clinically recognized concern in older adults; in addition to the considerable safety concerns of adverse drug-drug interactions, routine adherence to medications decreases as total pill burden increases [19]. Additionally, there is a substantial financial investment for individuals using DSs, who pay into the multi-billion dollar DS industry in the hopes of improving their health. Meanwhile, the benefits of DS use remain questionable, at best. Findings from a recent pharmacokinetic study suggested that supplement use can contribute to anticholinergic burden, and potentially contribute to adverse cognitive effects [20]. Two analyses from the RCT COSMOS study suggested that multivitamin use may improve cognition [21] and slow cognitive decline [22], but this is contrary to the findings of other studies [23]. In another RCT, the Vitamin D and Omega-3 Trial (VITAL) [24] questioned the use of Vitamin D and Omega-3 formulation agents from cardiovascular and cancer prevention to fall risk [25] and osteoporosis management [26]; yet, a recent cohort study found decreased dementia in those taking Vitamin D [27]. These conflicts highlight much of the clinical uncertainty, confusion, and interest surrounding the use of dietary supplements.

Multivitamins, Vitamin D and Omega-3 fatty acids were the most reported DS in our study. These findings are similar to those reported in cross sectional studies of older adults [3, 10, 12], but unique in that we have showcased how these trends changed across time in a stable cohort of older adult drivers. Our study included systematic separation of DS formulations into their individual DS components, revealing a significant degree of redundancy among DS users, particularly for vitamin D and multivitamin formulations. It additionally demonstrates that people who take a component in the “Other” category (most commonly herbal compounds) are likely to be taking two or more. Interestingly, individual supplements which are under study for having more potential for physiologic effect, such as red yeast, cannabis products, and cocoa, had relatively infrequent use in this study population. Turmeric/curcumin compounds were the most common herbal supplement, with a significant increase in use over time. Apart from turmeric, there was lower-than-expected use of many of these common herbal supplements.

Strengths of our study include the use of ‘brown-bag’ review, the longitudinal assessment in this stable cohort, and the ability to contrast supplements and prescribed (and known clinically relevant) medications. This highlights a ‘real world’ assessment of the frequency of both the use of over-the-counter supplement agents in older adults, but also the burden of their contribution to the total consumed pills/pharmacologic agents.

The authors recognize limitations to this analysis. While the use of brown-bag review allowed for more comprehensive evaluation of medication and DS use, this annual review did not assess for regular and consistent use, such as day-to-day compliance. Additionally, supplements used and stopped between the annual reviews were not captured. Heterogeneity in dietary supplements as a whole lack of oversight/consistency, and a subsequent error rate in supplement and supplement component identification (particularly with certain groups such as multivitamins) also limited analysis of supplement-specific effects and outcomes. Finally, the study population was largely high functioning older adult drivers who represent a higher education and income level, living in urban and suburban areas (with the exception of a single study location that included many rural participants, Cooperstown NY), which may limit generalization of these findings to other community dwelling older adults.

Given the frequency and burden of dietary supplement use, future studies are clearly needed, which should include supplement-supplement, supplement-medications, and supplement-health interactions/implications. Attention is needed to focus clinical and research efforts on these extremely common and potentially physiologically active compounds.

## Conclusion

More than 80% of older adults took at least one DS over the 2-year study period, with nearly two-thirds taking at least one supplement at any given time point, and just over half continually reporting use. Older adults who were White, female, older, and on multiple non-DS medications took a higher mean number of supplements; use increased with age. Vitamin D, Multivitamins, and Omega-3 formulations were the most common supplements. These findings showcase the substantial burden of DS use in older adult populations, and further attention is needed to better understand benefits, costs, and interactions with health and polypharmacy.

### Electronic supplementary material

Below is the link to the electronic supplementary material.

Supplementary Material 1

Supplementary Material 2

## Data Availability

The data that support the findings of this study are available from AAA LongROAD Foundation and the corresponding study team; restrictions apply to the availability of these data, which were used under approval for the current study, and so are not publicly available. Data are however available from the authors upon reasonable request and with permission of the AAA LongROAD study team.

## Abbreviations

AHFS: American Society of Health-System Pharmacists -
DS: Dietary Supplement
LongROAD: AAA Longitudinal Research on Aging Drivers
