# Supplementary material for: Dietary supplement use is common in older adult drivers: an analysis from the AAA LongROAD study
Source: BMC Complement Med Ther. 2024 Aug 30;24:319. doi: 10.1186/s12906-024-04623-x (PMC11363526; doi:10.1186/s12906-024-04623-x)
Supplement: Supplementary file 1 — Supplementary Material 1 [file 12906_2024_4623_MOESM1_ESM.docx]

| **Table 6 (suggested Appendix 1) - List of top 90 aggregate dietary supplements (DS) in the AAA LongROAD study database.*** | | | | | |
| --- | --- | --- | --- | --- | --- |
| Vitamin D | Vitamin E | Vitamin B6 | MSM | Chondroitin/ Glucosamine/ MSM/ Vitamin D | Vitamin B3 |
| Omega 3 | Artificial Tears | Cinnamon | Mushroom | Ginseng | Coenzyme Q/ Omega 3 |
| Calcium/ Vitamin D | Vitamin B9 | Resveratrol | B Combo/ Vitamin C | Calcium/ Potassium/ Vitamin D | Omega 3/ Vitamin D |
| Other | Vitamin B7 | Digestive Enzymes | Chondroitin | Lecithin | Chondroitin/ Glucosamine/ Vitamin C |
| Vitamin C | Glucosamine | Vitamin A | DHEA | B2 | Cocoa Flavanols |
| Calcium | Melatonin | Lipoic Acid | Calcium/ Magnesium/ Vitamin D | Cranberry/ Vitamin C | Coenzyme Q/ Vitamin E |
| B12 | Undetermined | Vitamin K | Calcium/ Magnesium/ Vitamin D/ Zinc | Vitamin D/ Vitamin K | Vitamin C/ Vitamin E |
| Coenzyme Q | Flaxseed | Red Yeast | Vitamin B1 | Capsaicin | Calcium/ Magnesium/ Potassium |
| Eye Vitamin | Amino Acid | Milk Thistle | Cannabis Product | Collagen | Vitamin C/ Vitamin D |
| Magnesium | Zinc | Chondroitin/Glucosamine/ MSM | Ginger | Vitamin A/ Vitamin D | Magnesium/ Potassium |
| B Combo | Cranberry | Calcium/ Magnesium/ Zinc | Grape Seed | Chondroitin/ Glucosamine/ Vitamin D | Vitamin C/ Zinc |
| Chondroitin/ Glucosamine | Calcium/ Magnesium | Elemental Mineral | Aloe Vera | Glucosamine/ Vitamin D | B Combo/ Omega 3 |
| Probiotic | Saw Palmetto | Ginkgo Biloba | St John’s Wort | Omega 3/ Flaxseed | Vitamin B12/ Vitamin B9 |
| Potassium | Garlic | Vitamin C/ Rose Hips | Echinacea | Calcium/ Vitamin C | Vitamin B6/ Zinc |
| Turmeric | Topical Products | Glucosamine/ MSM | Apple Cider Vinegar | Omega 3/ Vitamin E | Vitamin B7/ Vitamin C/ Vitamin E |

* The Remaining supplements (not shown) have three or fewer instances each over the study period.
