# Supplementary material for: Dietary supplement use is common in older adult drivers: an analysis from the AAA LongROAD study
Source: BMC Complement Med Ther. 2024 Aug 30;24:319. doi: 10.1186/s12906-024-04623-x (PMC11363526; doi:10.1186/s12906-024-04623-x)
Supplement: Supplementary file 2 — Supplementary Material 2 [file 12906_2024_4623_MOESM2_ESM.docx]

| **Table 7 (suggested Appendix 2) - Complete list of separated/individual dietary supplement categories in the AAA LongROAD study database (n=59)** | | | | |
| --- | --- | --- | --- | --- |
| Apple Cider Vinegar | B Combo | Echinacea | Melatonin | Saw Palmetto |
| Aloe Vera | Calcium | Elem. Mineral | Magnesium | St John’s Wort |
| Amino Acid | Cannabis | Eye Vitamin | Milk Thistle | Topical |
| Artificial Tears | Capsaicin | Flaxseed | MSM | Turmeric |
| Vitamin B1 | Chondroitin | Garlic | Mushroom | Undetermined |
| Vitamin B12 | Cinnamon | Ginger | Multivitamin | Vitamin A |
| Vitamin B2 | Cocoa | Ginkgo Biloba | Omega3 | Vitamin C |
| Vitamin B3 | Coenzyme Q | Ginseng | Other | Vitamin D |
| Vitamin B5 | Collagen | Glucosamine | Potassium | Vitamin E |
| Vitamin B6 | Cranberry | Grapeseed | Probiotic | Vitamin K |
| Vitamin B7 | DHEA | Lecithin | Red Yeast | Zinc |
| Vitamin B9 | Digestive Enzymes | Lipoic Acid | Resveratrol | --- |
